# Prevalence dependent calibration of a predictive model for nasal carriage of methicillin-resistant *Staphylococcus aureus*

**DOI:** 10.1186/1471-2334-13-111

**Published:** 2013-02-28

**Authors:** Johannes Elias, Peter U Heuschmann, Corinna Schmitt, Frithjof Eckhardt, Hartmut Boehm, Sebastian Maier, Annette Kolb-Mäurer, Hubertus Riedmiller, Wolfgang Müllges, Christoph Weisser, Christian Wunder, Matthias Frosch, Ulrich Vogel

**Affiliations:** 1 Institute for Hygiene and Microbiology, University of Würzburg, Josef Schneider-Strasse 2, Würzburg, 97080, Germany; 2 Institute for Clinical Epidemiology and Biometrics, University of Würzburg; Center for Clinical Studies, University Hospital Würzburg, Josef Schneider-Strasse 2, Würzburg, 97080, Germany; 3 Service Center for Medical Informatics, University Hospital Würzburg, Josef Schneider-Strasse 2, Würzburg, 97080, Germany; 4 Department of Maxillofacial Surgery, University Hospital Würzburg, Pleicherwall 2, Würzburg, 97070, Germany; 5 Department of Medicine I, University Hospital Würzburg, Oberduerrbacher Strasse 6, Würzburg, 97080, Germany; 6 Department of Dermatology, University Hospital Würzburg, Josef Schneider-Strasse 2, Würzburg, 97080, Germany; 7 Department of Urology and Paediatric Urology, University Hospital Würzburg, Oberduerrbacher Strasse 6, Würzburg, 97080, Germany; 8 Department of Neurology, University Hospital Würzburg, Josef Schneider-Strasse 11, Würzburg, 97080, Germany; 9 Department of Sugery II, University Hospital Würzburg, Oberduerrbacher Strasse 6, Würzburg, 97080, Germany; 10 Department of Anesthesiology, University Hospital Würzburg, Oberduerrbacher Strasse 6, Würzburg, 97080, Germany; 11 Present address: Hannover Medical School, Institute of Virology, Carl Neuberg-Strasse 1, Hannover, 30625, Germany

**Keywords:** Methicillin-resistant staphylococcus aureus, Infection control, Clinical prediction rule, Predictive value of tests, False positive reactions, Calibration

## Abstract

**Background:**

Published models predicting nasal colonization with Methicillin-resistant *Staphylococcus aureus* among hospital admissions predominantly focus on separation of carriers from non-carriers and are frequently evaluated using measures of discrimination. In contrast, accurate estimation of carriage probability, which may inform decisions regarding treatment and infection control, is rarely assessed. Furthermore, no published models adjust for MRSA prevalence.

**Methods:**

Using logistic regression, a scoring system (values from 0 to 200) predicting nasal carriage of MRSA was created using a derivation cohort of 3091 individuals admitted to a European tertiary referral center between July 2007 and March 2008. The expected positive predictive value of a rapid diagnostic test (GeneOhm, Becton & Dickinson Co.) was modeled using non-linear regression according to score. Models were validated on a second cohort from the same hospital consisting of 2043 patients admitted between August 2008 and January 2012. Our suggested correction score for prevalence was proportional to the log-transformed odds ratio between cohorts. Calibration before and after correction, i.e. accurate classification into arbitrary strata, was assessed with the Hosmer-Lemeshow-Test.

**Results:**

Treating culture as reference, the rapid diagnostic test had positive predictive values of 64.8% and 54.0% in derivation and internal validation corhorts with prevalences of 2.3% and 1.7%, respectively. In addition to low prevalence, low positive predictive values were due to high proportion (> 66%) of *mecA*-negative *Staphylococcus aureus* among false positive results. Age, nursing home residence, admission through the medical emergency department, and ICD-10-GM admission diagnoses starting with “A” or “J” were associated with MRSA carriage and were thus included in the scoring system, which showed good calibration in predicting probability of carriage and the rapid diagnostic test’s expected positive predictive value. Calibration for both probability of carriage and expected positive predictive value in the internal validation cohort was improved by applying the correction score.

**Conclusions:**

Given a set of patient parameters, the presented models accurately predict a) probability of nasal carriage of MRSA and b) a rapid diagnostic test’s expected positive predictive value. While the former can inform decisions regarding empiric antibiotic treatment and infection control, the latter can influence choice of screening method.

## Background

*Staphylococcus aureus* can cause a wide range of hospital and community associated infections including endocarditis, pneumonia, osteomyelitis, and wound infections. In contrast to methicillin-susceptible *S. aureus* (MSSA), infections by methicillin-resistant *S. aureus* (MRSA) were described as causing increased length of hospital stay [1], higher lethality [2], and delayed resolution of ventilator-associated pneumonia despite appropriate antibiotic treatment [3]. In the healthcare setting, yearly over 150,000 individuals are estimated to suffer from infections by MRSA in the European Union, with attributable costs for hospitals exceeding 380 million Euro [4]. In the United States, over 90,000 invasive infections are estimated to occur per year, translating to an incidence rate of over 30/100,000 [5].

The spectrum of possible control measures aimed at curbing spread of MRSA include screening of patients for nasal carriage on admission, isolation of case patients, decolonization at the end of hospitalization, screening of contacts and hospital staff for colonization, ward closures in case of exuberant nosocomial transmissions [6], and antibiotic prescribing interventions [7]. Several countries including the Netherlands [8] and, since 2006, the UK [9] recommend screening of hospital admissions. A meta-analysis focusing on the use of rapid molecular tests showed that screening at admission reduced incidence rates of bacteremia, yet not surgical-site infections by MRSA [10].

Many models predicting carriage of MRSA focus on discrimination [11-14], i.e. the ability to separate individuals colonized with MRSA from the rest by giving a yes/no answer for a given set of patient characteristics. A reason for this may be the interest of researchers to restrict screening efforts to a subset of individuals with high risk, thereby saving diagnostic resources. This approach is recommended in low prevalence areas [15] and has been adopted in countries including the Netherlands [8]. Nevertheless, sensitivities of prediction tools in separating carriers from non-carriers are moderate at best: e.g. Robicsek et al. presented models with comparably good discriminatory ability, which nevertheless missed at least 32% of carriers within the validation cohort even if 30% of admissions were screened [11]. Similarly, other models would have missed approximately 20% [14] and 14% [12] of carriers even if larger proportions (> 62%) of admissions had been screened, respectively. Despite limited discriminatory ability and versatility of tools focusing on providing binary results (by suggesting who to screen or not to screen), very few published models address calibration, i.e. the ability to predict individual probability of carriage. One example is a model by Sax et al., which, however, assessed calibration only across three risk strata [13].

Currently, no published models adjust for the effect of prevalence. Even though formally not affecting overall measures of discrimination such as AUC in ROC-analysis, prevalence affects cutoff and threshold values extractable from predictive models, and thus accurate classification into two or more strata across settings with different rates of MRSA. In addition, it has a profound impact on the positive predictive values (PPV) of rapid molecular screening tests. Multiple reports using tests based on the amplification of fragments spanning the 3^′^ end of SCC*mec* and the 5^′^ end of *orfX* document low PPVs between 31% to 65% in settings with prevalence below 5%, regardless of the test’s producer [16-18].

In the present study we derive and internally validate a scoring system able to predict the probability of nasal MRSA carriage and the expected PPV of a rapid diagnostic test in individuals admitted to a European tertiary referral center. Furthermore, we propose and internally validate a correction method for prevalence differing from our derivation cohort, thus facilitating use of the presented models in settings with different or changing rates of MRSA.

## Methods

### Patient cohorts

We used two cohorts of patients admitted to the University Hospital of Wuerzburg, which is a tertiary referral center with approximately 50,000 inpatient admissions per year. The first cohort (derivation cohort, DC) consisted of a study approved by the Ethics Committee of the Medical Faculty of the University of Würzburg (reference no. 62/07; applicant: UV) aiming to estimate the prevalence of MRSA nasal carriage in admissions to 13 wards and admissions passing through the medical emergency department between July 2007 and March 2008. Wards were selected to ensure a representation of surgical, medical, intensive care, and non-intensive care patients. All swabbed individuals or their legal guardians gave verbal consent to being tested; this policy was additionally approved by the legal department of the University Hospital of Wuerzburg. Individuals were included into this cohort if they were at least 18 years old and a rapid molecular test had been performed from a nasal swab no later than 2 days after admission. Of patients admitted more than once, only data and test results pertaining to the first admission were considered. In total, 3091 of 5039 eligible admitted individuals (61.3%) were included. Reasons for moderate coverage encompassed, besides failure to administer the test (1488 of 5039, 29.5%), failure to swab the nose (310, 6.2%), and failure to test within 48 hours after admission (150, 3.0%). The age range was 18 to 102 (interquartile range: 46 to 75); mean and median ages were 59.7 and 64.0 years, respectively. Men slightly prevailed in the cohort with 54.9%. As one of the authors requested the Ethics committee’s vote (UV) and two authors (UV, JE) are part of the hospital’s infection control team assigned with the implementation of risk based screening, no additional permission was necessary to access and use data from the derivation cohort. The second cohort (internal validation cohort, IVC) consisted of a convenience sample of admissions to units F, K, L, and M (Table 1), which voluntarily continued non-selective screening with the rapid molecular test after March 2008. As above, all patients or their legal guardians consented to being tested for the presence of MRSA. For this cohort, no separate approval by the Ethics committee was sought, because it consisted of routinely collected data from the laboratory of the Institute for Hygiene and Microbiology and the hospital information system required for the implementation of a German national recommendation to perform risk based screening [19]. In order to comply with these recommendations, above wards pilot-tested an electronic nursing history system at various intervals between August 2008 and January 2012, which also recorded information regarding nursing home referral. The electronic nursing history was available for 2978 of 6449 admissions ≥ 18 years old within this period; 2509 of these were tested with nasal swabs, yet 466 were excluded because swabs were performed > 48 hours after admission. Thus, 2043 patients (68.6% of individuals with electronic nursing history) were included into IVC. Analogous to DC, only data from the first admission were included into IVC for patients admitted more than once. The age range in IVC was 18 to 99 years (interquartile range: 56 to 78); mean and median ages were higher than in DC with 65.8 and 70.0 years, respectively (p < 0.001, Mann–Whitney test). The proportion of men in IVC was 54.1% and not significantly different from DC (p = 0.586, Chi-squared test). Only anonymized data from DC and IVC were stored and used for analysis at all times.

**Table 1:** List of units participating in the derivation cohort (DC) between July 2007 and March 2008

**Unit(s)**	**Specialty**	**ICU**	**MRSA neg**	**MRSA pos**	**Prevalence**
A	Medicine		191	9	4,5
B, D	Surgery		501	10	2,0
C	Surgery	+	134	4	2,9
E	Urology		308	1	0,3
***F***	Maxillofacial Surgery		477	16	3,2
G, H, I, J	Dermatology		409	9	2,2
***K***	Neurology		358	3	0,8
***L***	Neurology	+	156	3	1,9
***M***	Anesthesiology	+	162	0	0,0
N	Emergency Medicine		323	17	5,0
All			3019	72	2,3

Units also providing patients for the validation cohort between Auguts 2008 and January 2012 are bold italic. ICU: intensive care unit.

### Acquisition of data

For DC, patient related data including age, sex, referral from another hospital, 10^th^ version of the German Modification of the International Classification of Diseases’ (ICD-10-GM) code of admission diagnosis, date of admission, ward, and admission via medical emergency department were extracted prospectively from the hospital information system. Information regarding nursing home referral was collected prospectively using a paper form attached to the laboratory request slip accompanying the nasal swab. For IVC, data were extracted retrospectively from the hospital information system and the new electronic nursing history system.

### Rapid diagnostic test and confirmation of methicillin resistance

The GeneOhm test (BDGO, Becton Dickinson GmbH, Heidelberg, Germany) was used to detect MRSA in nasal swabs. All tests were compared to culture, which was treated as the reference standard. Thus, after use for rapid testing, sampling buffers were overlaid with brain-heart infusion (BHI) broth, incubated at 36°C overnight, and plated on Columbia agar with 5% sheep blood, Difco^TM^ Baird-Parker (Becton Dickinson GmbH), and CHROMagar^TM^ MRSA medium (CHROMagar, Paris, France). Solid media were subsequently incubated for 48 h at 36°C with 5% CO_2_. Mauve colonies on Chromagar, or colonies suggestive of *S. aureus* on blood or Baird-Parker medium were isolated and confirmed with Vitek II (bioMérieux, Nürtingen, Germany) using GP and AST-P592 cards for species identification and antimicrobial susceptibility testing, respectively. Presence of the *mecA* gene was confirmed by PCR using primers MecA1 and MecA2 published previously [20]. Each rapid test result was grouped into one of five mutually exclusive categories: a) true positive (tp), if a positive test result was confirmed by culture of MRSA; b) deletion variant (dv), if a positive test result was followed by culture of methicillin-susceptible *S. aureus*; c) not culturally confirmed (nc), if a positive test result was not followed by growth of *S. aureus* (i.e. positive result, but not “dv” and not “tp”); d) false negative (fn), if a negative result was followed by growth of MRSA; and e) true negative (tn), if a negative result was not followed by growth of MRSA. Categories “dv” and “nc” were both regarded as false positives (fp). As only a single result was considered per admission even if multiple rapid tests had been processed, inclusion of results was prioritized according to the hierarchy tp > dv > fn > nc > tn. For patients admitted more than once, data from the first admission only were included into analyses.

### Statistical analyses

Cultural results were treated as the gold standard for the estimation of the rapid diagnostic test’s (RDT) performance parameters, i.e. specificity (Sp), sensitivity (Sn), positive predictive value (PPV), and negative predictive value (NPV). Ninety-five percent confidence intervals for the test’s performance parameters were calculated assuming a binomial distribution of estimates. Differences in proportions and performance parameters between cohorts were assessed with the Fisher’s Exact Test. Multivariate logistic regression testing association of covariates, which were all treated as exposures, with carrier status was performed with the program R [21]. Predictors were removed from the model, unless this significantly increased deviance of the model. Similarly, levels of categorical variables (e.g. admission diagnoses) were allowed to aggregate, if this did not significantly increase deviance of the model. In order to facilitate the model’s use in the clinical setting, log-transformed odds were translated to scores ranging from 0 to 200. Score translation and ROC analysis was accomplished using the R-package “rms” [22]. Briefly, the score (denoted as *S*) represents a linear transformation of fitted log-transformed odds (denoted as *L*) of the form *S=a+b L*, whereby parameters *a* and *b* are chosen to ensure optimal representation of *L* on a scale ranging from 0 to 200; transformation of *S* to individual probability of carriage *p* was carried out using the formula $p={\left(e^{\frac{a-S}{b}}+1\right)}^{-1}$. Prediction of the RDT’s positive predictive value (PPV) from the score was performed using non-linear regression based on the function $\mathrm{PPV}={\left(\frac{f}{o}+1\right)}^{-1}$, where odds $o=e^{\frac{S-a}{b}}$, and *f*, equaling $\frac{1-\mathrm{Sp}}{\mathrm{Sn}}$, was the parameter to be estimated. The suggested correction score *S*_*c*_ added according to prevalence was computed using the formula *S*_*c*_ = log(*OR*) · *b*; here, *OR* represents the odds ratio between the new population being corrected for and the derivation cohort, calculated as $\mathrm{OR}=\frac{\mathrm{odd}s_{\mathrm{new}}}{0.0238}$; moreover, *b* is the slope of the linear transformation between log-transformed odds and score (see above). Goodness-of-fit of the predictive models was assessed with the Hosmer-Lemeshow-Test [23].

## Results

### Performance of the rapid diagnostic test

Of 3091 tests considered in the first cohort (DC), 70, 38, 2981, and 2 were categorized as true positive, false positive, true negative, and false negative, respectively. Prevalence of culturally confirmed MRSA nasal carriage within DC was 2.3% (Table 1). Of 38 false positives, 25 (65.8%) were due to methicillin susceptible *S. aureus* that lacked *mecA*, as shown by PCR. Retesting of these strains, designated “deletion variants”, with BDGO yielded positive results, suggesting remain of DNA spanning a part of the SCC*mec* cassette and *orfX*. The test’s sensitivity, specificity, PPV, and NPV in DC was 97.2% (95% confidence interval 90.3% to 99.7%), 98.7% (98.2% to 99.1%), 64.8% (55.0 to 73.8%), and 99.9% (99.8% to 100%), respectively. In the second cohort (IVC) 34, 29, 1980, and 0 were true positive, false positive, true negative, and false negative, respectively; MRSA prevalence in IVC was 1.7%. Of 29 false positive results, 23 (79.3%) were due to deletion variants. Sensitivity, specificity, PPV, and NPV in IVC were 100% (89.7% to 100%), 98.6% (97.9% to 99.0%), 54.0% (40.9% to 66.6%), and 100% (99.8% to 100%), respectively. Differences of performance parameters were not significantly different between cohorts according to Fisher’s Exact Test. Also, the difference in prevalence between DC and IVC was not statistically significant (p = 0.109, Fisher’s Exact Test).

### Prediction of nasal carriage of MRSA

**Figure 1:**
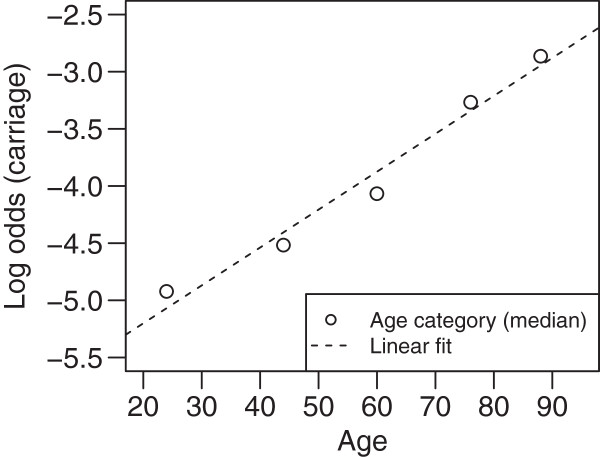
Graphical representation of the linear relationship between age and log-transformed odds of carriage within the derivation cohort according to univariate logistic regression.

**Figure 2:**
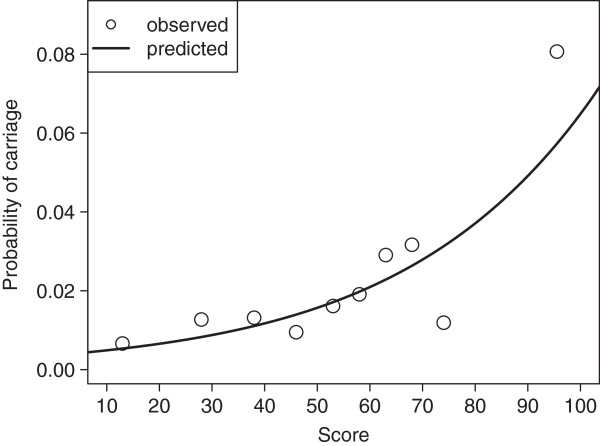
**Fit of logistic regression model in the prediction of carriage probability in the derivation cohort.** Open circles labeled “observed” represent probability within intervals represented by median score. Interval limits were chosen to ensure equal number of individuals per interval.

Due to the linear relationship of log-transformed odds of nasal carriage and age within DC (Figure 1), we treated age as a continuous variable in our models. We evaluated exposures including age, sex, referral from another hospital, referral from a nursing home, admission via the emergency department, and the first letter of the admission diagnosis according to ICD-10-GM. We allowed strata of categorical variables (e.g. admission diagnoses) to lump to simpler forms if coefficients of individual levels were similar and aggregation did not increase the deviance of the model. Age, admission via emergency department, aggregation of admission diagnoses starting with “A” (“Certain infectious and parasitic diseases”) or “J” (“Diseases of the respiratory system”), and nursing home referral were significantly associated with carrier status (Table 2). The group of admission diagnoses starting with letters “A” or “J” comprised 40 distinct ICD-10-GM codes assigned to a subset of 84 individuals; the five most common diagnoses A46 (“Erysipelas”, 18 of 84), J18.8 (“Other pneumonia, organism unspecified”, 10/84), A41.9 (“Sepsis, unspecified”, 5/84), J44.9 (“Chronic obstructive pulmonary disease, unspecified”, 5/84), and J32.0 (“Chronic maxillary sinusitis”, 4/84) were found in 50% of patients (42/84) within this group. In contrast to the average prevalence (2.3%), rates of MRSA carriage among medical emergency admissions, nursing home residents, and patients with admission diagnoses starting with “A” or “J” were 5.0% (17/340), 11.0% (11/100), and 9.5% (8/84), respectively. Based on this model, we transformed the fitted log-transformed odds to a score ranging from 0 to 200 using the intercept *a* = 190.7 and linear coefficient *b* = 34.0. The score for a patient in DC was computed by subtracting 10 from the age and adding to this number 22 points in case of emergency admission, 37 points, if the patient is nursing-home resident, and 39 points, if the admission diagnosis belonged to headings “A” or “J” according to ICD-10-GM (Table 2). The model approximated observed score-based probability reasonably well (Figure 2), as confirmed by the Hosmer-Lemeshow-Test (χ^2^ = 7.9, 8 degrees of freedom, p=0.440). Further, the model had moderate discriminatory ability with an AUC of 0.693 according to ROC-analysis.

**Table 2:** Odds ratios (OR) from the logistic regression model with 95% confidence intervals (CI) used for the prediction of the probability of nasal carriage of Methicillin-resistant Staphylococcus aureus

**Variable**	**Crude OR (95% CI)**	**Adjusted OR (95% CI)**	**p**_**W**_	**Score**
Age	1.04 (1.02,1.05)	1.03 (1.01,1.05)	< 0.001	Age - 10
Nursing home	5.94 (3.02,11.67)	3.00 (1.45,6.19)	0.003	37
Admission diagnosis: A or J	4.84 (2.24,10.45)	3.18 (1.39,7.28)	0.006	39
Emergency admission	2.58 (1.48,4.5)	1.93 (1.08,3.47)	0.028	22

P_w_: p-value according to the Wald-Test. An initial score for a patient is calculated by subtracting 10 from age and adding values in column “Score”, if the corresponding variable is true. If prevalence differs from 2.3%, a correction score (Figure 5) has to be added.

### Prediction of the rapid diagnostic test’s positive predictive value

**Figure 3:**
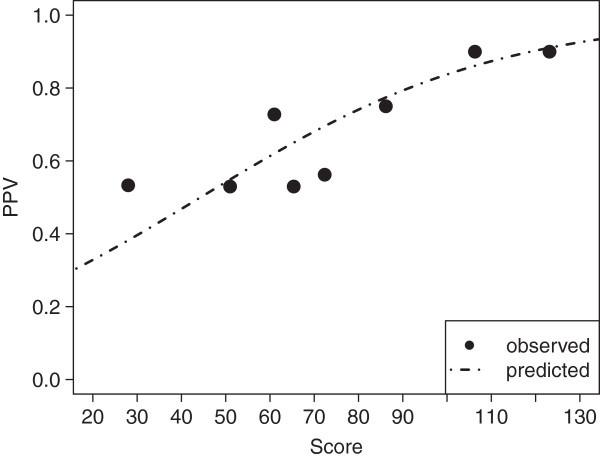
**Fit of non-linear regression model describing the rapid diagnostic test’s positive predictive value depending on the score within the derivation cohort.** Full circles labeled “observed” represent positive predictive value within intervals represented by median score. Interval limits were chosen to ensure equal number of positive results per interval.

Using non-linear regression, we fitted a function describing PPV’s dependence on specificity, sensitivity (the latter two values merged within the parameter *f*), and individual odds, whereby observed PPV was aggregated into 8 groups by score (Figure 3). The fitted parameter *f* was 0.0135, and thus very close to the observed quotient $\frac{1-\mathrm{Sp}}{\mathrm{Sn}}$ (0.0134) within DC. The Hosmer-Lemeshow-Test on a subset of DC with positive molecular results did not show evidence for insufficient fit (χ^2^ = 9.4, 6 d.f., p=0.150).

### Internal validation and calibration

**Figure 4:**
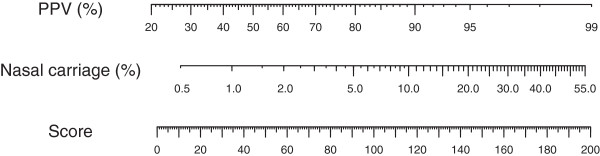
Nomogram describing functional relationship between score, probability of nasal carriage of Methicillin-resistant Staphylococcus aureus, and the rapid diagnostic test’s positive predictive value.

**Figure 5:**
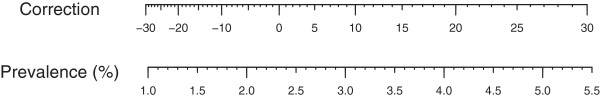
Nomogram depicting relationship between prevalence and point values to be added to the score as an adjustment for prevalence.

**Figure 6:**
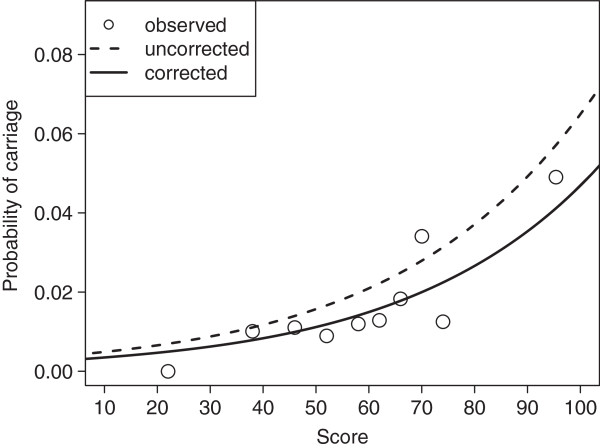
**Fit of predictive model in the internal validation cohort with and without correction.** Open circles labeled “observed” represent probabilities of carriage within intervals represented by median score. Interval limits were chosen to ensure equal number of individuals per interval.

**Figure 7:**
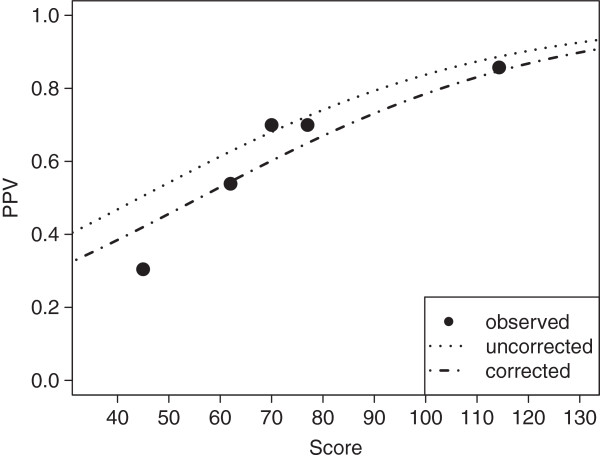
**Fit of model predicting the rapid diagnostic test’s positive predictive value in the validation cohort.** Full circles labeled “observed” represent positive predictive values within intervals represented by median score. Interval limits were chosen to ensure equal number of positive results per interval.

We internally validated the predictive ability of functions translating scores into individual probability of nasal carriage of MRSA and the PPV of the rapid diagnostic test (Figure 4) on a separate cohort from the same hospital (IVC). Although the difference in prevalences between DC and IVC did not reach statistical significance (1.7% vs. 2.3%, p = 0.109, see above), we explored whether a correction for this disparity resulted in a more accurate prediction. For IVC, score correction was equivalent to −11.7 points (Figure 5); addition of this value led to a better graphical fit of score-based prediction (Figure 6). This was further indicated by reduction of the χ^2^ value of the Hosmer-Lemeshow-Test from the uncorrected (χ^2^ = 8.1, d.f. = 8, p = 0.427) to the corrected (χ^2^ = 4.3, 8 d.f., p = 0.831) model, although in both cases the fit was acceptable (demonstrated by p > 0.05). Moreover, categorizing patients into arbitrary strata with “very low” (≤ 1%), “low” (> 1% to ≤ 3%), “intermediate” (> 3% to ≤ 6%), and “high” (> 6%) probability of carriage was only accurately achieved with the corrected model (Table 3); specifically, the uncorrected model classified 299 patients into the “intermediate” category, although their observed probability was 1.7% (instead of > 3%). In contrast, the corrected model shifted 261 of “intermediate” patients into category “low”, thus achieving accurate prediction. In total, correction led to a reclassification of 561 (27.5%) individuals. Similarly, correction led to a better fit of the model predicting PPV (Figure 7) according to the Hosmer-Lemeshow-Test (uncorrected: χ^2^ = 4.5, d.f. = 3, p = 0.211; corrected: χ^2^ = 2.1, d.f. = 3, p = 0.558). This was also evident from the reclassification table (Table 4), where the ability to classify individuals into arbitrary groups with “very low” (≤ 40%), “low” (> 40% to ≤ 60%), “intermediate” (> 60% to ≤ 80%), and “high” (> 80%) PPVs was compared: the uncorrected model slightly overestimated PPVs in the “low” and “intermediate” groups, where observed PPVs were 40% (instead of > 40%) and 60% (instead of > 60%), respectively. After correction 835 (40.9%) individuals were reclassified; the new classification accurately reflected observed PPVs within the pre-specified strata (Table 4).

**Table 3:** Classification into probability intervals within validation cohort according to uncorrected and corrected scores

		**Corrected**				
		**[0,1]**	**(1,3]**	**(3,6]**	**(6,100]**	**Observed**
**uncorr.**	**[0,1]**	237	0	0	0	0.0 (0/237)
	**(1,3]**	261	1149	0	0	1.5 (21/1410)
	**(3,6]**	0	261	38	0	1.7 (5/299)
	**(6,100]**	0	0	39	58	8.2 (8/97)
	**observed**	0.4 (2/498)	1.7 (24/1410)	5.2 (4/77)	6.9 (4/58)	

Round and square brackets specify values excluded or included, respectively, from the interval.

**Table 4:** Prediction of intervals of a rapid diagnostic test’s expected positive predictive value within the validation cohort according to uncorrected and corrected scores

		**Corrected**				
		**[0,40]**	**(40,60]**	**(60,80]**	**(80,100]**	**Observed**
**uncorr.**	**[0,40]**	173	0	0	0	0.0 (0/4)
	**(40,60]**	226	520	0	0	40.0 (8/20)
	**(60,80]**	0	584	433	0	60.0 (18/30)
	**(80,100]**	0	0	25	82	88.9 (8/9)
	**observed**	25.0 (2/8)	47.1 (16/34)	71.4 (10/14)	85.7 (6/7)	

Round and square brackets specify values excluded or included, respectively, from the interval. Values in parentheses in row and column labeled “observed” represent quotients of true positives divided by all positives within the interval.

## Discussion

We describe the derivation and internal validation of a scoring system able to predict the probability of nasal MRSA carriage and the expected PPV of a molecular screening test in admissions to a European tertiary referral center with low prevalence. In the assessment of admitted patients published models mainly focus on measures of discrimination, which summarize the ability to separate true positive from negative results. Many models assessed for their discriminatory ability have been created with the primary aim of restricting screening efforts to a subset of hospital admissions. Nevertheless, it seems questionable, if predictive models can attain high degrees of separation into carriers and non-carriers at all, as even elaborate models fail to display acceptable discrimination and thus sensitivity in detecting unknown carriers when screening is restricted to a subset of the admitted population. E.g. a recently published model with comparably good discriminatory ability (AUC 0.72 in ROC analysis) would have missed 32% of carriers even if 30% of admissions had been screened [11]. This seems to be an inherent limitation of prediction tools, since attainable AUCs depend on the distribution of risk in the population under study; specifically, only rarely encountered distributions displaying unusually wide spread, such as the uniform or u-formed distributions, allow AUCs (and with it discriminatory ability) to surpass values of 0.80 [24]. Given this imperfection, it seems futile to continue to design prediction tools for the sole purpose of suggesting who to screen on admission.

In addition to discrimination, however, models can be used for accurate prediction of disease probability, which is of primary clinical interest for the management or prevention of disease [24]. As an example, the European guidelines on cardiovascular disease prevention stress the importance of absolute risk for the planning of preventive measures [25]. Similarly, decisions regarding treatment, e.g. the choice of antibiotic before availability of cultural results in the presence of clinical infection, or infection control, e.g. choice of screening method or preliminary measures such as isolation, could be informed by predicted probability of MRSA carriage.

Using a set of potentially readily available predictors we established a predictive model and transformed its output into a score ranging from 0 to 200, whereby predictors were assigned point values in keeping with the sizes of the adjusted coefficients derived from the logistic regression model (Table 2). Unlike published models including factors such as previous antibiotic consumption [11-14,26,27], we have restricted predictors to a set of unequivocal variables potentially extractable from the hospital information system on the day of admission. The advantage of this strategy is that risk assessment would not depend on additional clinician input, who may have competing priorities, particularly in busy services [28]. Several authors identified similar risk factors to the ones in our model including age [11,14,29-33], nursing home residence [11,14,34,35], and emergency admission [30,36]. Comparable to the group of admission diagnoses under the ICD-10-GM headings “A” (“Certain infections and parasitic diseases”) or “J” (“Diseases of the respiratory system”), other authors found certain infections including pneumonia and wound infections [30], skin and soft tissue infections [26], skin and bone infection [11], and lung disease [11] present at admission significantly associated with MRSA carrier status. Nevertheless, several groups failed to find an association between risk factors identified in this study and MRSA carriage; e.g. Sax et al. did not find a significant association to nursing home residence or age > 85 years [13], while Harbarth et al. reported no association to admission through the emergency department [14]. It is difficult to interpret the high prevalence in the medical emergency department within DC compared to the rest of the wards; while it refers patients to other wards, more than 96% of patients in DC and IVD did not pass through the medical emergency department. Therefore it seems unlikely, that it filtered MRSA carriers out before they arrived to other wards. In contrast, the particularly low rate in Anesthesiology within DC (0 of 162, Table 1; 95% CI 0.0% to 2.3% according to binomial distribution) could have been due to chance, as prevalence in this unit within IVC (1.6%) did not differ significantly. Further, we were unable to confirm an independent influence of sex reported by several groups [11,14]. These differences likely reflect the inclusion of comparably weak or setting-specific risk factors; e.g. the fact that, in the present study, medical emergency admission had the lowest odds ratio of 1.9 among categorical variables tested and a comparably high p-value of 0.03 (Table 2) may explain why it is rarely reported as independent predictor in other studies.

Our score showed a useful fit within the DC as shown graphically (Figure 2) and using the Hosmer-Lemeshow test on 10 strata. We validated our score on a separate cohort (IVC) admitted to wards F, K, L, and M between August 2008 and January 2012. The prevalence in IVC compared to DC was lower with 1.7% vs. 2.3%, respectively. The reason for the differing prevalence was mainly due to lower previous prevalence within wards F, K, L, and N within DC (1.9%, Table 1). The model’s calibration in IVC was acceptable as shown by the Hosmer-Lemeshow-Test on 10 strata, suggesting that the small difference in prevalence did not necessarily require correction of the score. Nevertheless, correction resulted in more accurate fit, evidenced by the lower χ^2^- value of the Hosmer-Lemeshow-Test (Figure 6). Furthermore, while both corrected and uncorrected scores enabled classification of individuals into strata of increasing carriage probability, categorization using corrected scores more accurately reflected actual probability (Table 3). The proposed correction therefore facilitates use of our models in settings with different prevalence and thus promotes external validation. While the presented score correction represents a novel application in the area of MRSA prediction, its concept is not new: e.g. adjustment to background rates in cardiovascular risk are commonly applied to achieve accurate prediction, as exemplified by the recalibrated cardiovascular risk chart for Germany [37].

Apart from its effect on calibration, prevalence differences greatly affect the PPV of rapid diagnostic tests. Similar to other reports [16-18] we confirm that a commercial molecular test based on the amplification of a fragment spanning SCC*mec* and *orfX* has low PPVs in a low prevalence setting. The substantial proportions (> 66%) of methicillin-susceptible *S. aureus* strains lacking *mecA* among false positives contributed to this phenomenon. Although the specificities of the test were almost equal with 98.7% and 98.6% for DC and IVC, respectively, a small difference in prevalence of 0.6% caused a sizeable drop of PPV from 64.8% (70 of 108) to 54.0% (34 of 63). As higher prevalence is associated with higher PPV of molecular tests [38], we explored whether individual probability of carriage showed a similar relationship. Using non-linear regression, we fitted a function corresponding to the mathematical description of PPV by specificity, sensitivity, and individual odds (represented by the score) to observed PPV in eight strata with different scores (Figure 3). According to the Hosmer-Lemeshow-Test on a subset of DC with positive molecular test results, the fit was found acceptable. We then validated this model on IVC using the Hosmer-Lemeshow-Test on five strata. The reduction of strata compared to DC was due to the reduced number of positive results in IVC (in keeping with lower prevalence and sample size), as we aimed for a comparable number of positive results in each stratum; for DC and IVC each stratum thus had an average of 13.5 (108/8) and 12.6 (63/5) positive results, respectively. The fit was found useful before correction, yet in analogy to prediction of probability, it was improved after adjustment for prevalence. This was also evident from the reclassification tables, which demonstrated a more accurate categorization into pre-specified strata. Hence, in addition to the prediction of probability of carriage, our model also predicted the expected PPV of a rapid test for a given individual.

The score can be derived in external settings from a set of patient parameters (containing information on age, nursing home residence, emergency admission, and admission diagnosis) in two steps: 1) an initial score is calculated by substracting 10 from the age and further adding scores according to column “Score” in Table 2; 2) if external MRSA prevalence differs from 2.3% (prevalence of DC), a correction score, derivable from the nomogram in Figure 5 according to prevalence, has to be added to the initial score. The resulting final score can then be translated into probability of carriage and expected PPV using the nomogram in Figure 4. Probability of carriage can inform several clinical decisions. Empiric treatment in case of e.g. wound infections could include antibiotics suitable against MRSA if high probability of carriage is likely (e.g. ≥ 6%); also, preemptive isolation or further control measures could be automatically carried out in these cases. Further, the prediction of PPV could help target molecular tests to individuals where equivocal results are unlikely. Clearly, the formulation of a PPV-threshold above which molecular tests should be applied is a matter of taste; we propose that rapid diagnostic testing be restricted to individuals with expected PPV over 80%, and that cultural screening be used for the rest of admissions. For IVC, this threshold would include only 4.0% of individuals.

Our study has several limitations. In contrast to reports with screening compliance exceeding 80% [11,13,14,29,30], only 61% of eligible patients had been screened correctly in our derivation cohort. This was due to the high number of patients that had to be excluded due to testing of sites other than the nose and tests taken more than 2 days after admission. While incomplete coverage of patients may have introduced a bias, the models generated from DC still performed favorably in a separate cohort (IVC). Also, the internal validation cohort consisted of a convenience sample retrospectively extracted from a subset of wards that coincidentally pilot-tested an electronic nursing history system during different periods after August 2008. The different sampling method may have decreased comparability between DC and IVC. Specifically, it remains unknown, why age was different between the two cohorts. Increased age in IVC may have been due to demographic changes within the admitted patients, as the proportion of males remained the same (even when compared to wards F, K, L, M in DC), and increase of age was not associated with increased prevalence, as might have been expected in the case of selective inclusion of older patients. The use of ICD-10-GM admission diagnoses might also have introduced a bias, since the degree of inter-rater agreement in coding admission diagnoses is not well established. Nevertheless, it is conceivable that agreement on ICD-10 chapter headings, as used in our study, will be reasonably high even between raters in different countries. Also, availability of coded admission diagnoses within the first day of admission may pose a hindrance for the use of our models: in our center, only 49% and 66% of admission diagnoses were available in IVC as ICD-10-GM codes on the day of admission and one day after admission, respectively; thus, speedy coding of admission diagnoses (alternatively of chapter headings) has to be implemented for the automated use of our models. Nevertheless, as the most authorative classification of diseases, the choice of ICD-10-GM seems natural; further research could elucidate, whether codes including U80.0 (*S. aureus* with methicillin or other resistance), Z22.3 (carrier of staphylococci or other bacteria), or Z29.0 (isolation) predicts MRSA carriage. While neither diagnosis was coded as admission diagnosis in our cohorts, the previous use of these codes as secondary diagnoses may well be predictive; nevertheless, this would require that coding data from other hospitals be made available to the admitting center. Finally, our sample size was rather small for a low prevalence area with 3091 and 2043 individuals, respectively; this likely decreased our power to find weak associations between predictors and MRSA carriage. Despite these limitations, we were able to derive and internally validate a scoring system that can be used both for the prediction of individual probability of carriage and the expected positive predictive value of a rapid diagnostic test. Due to the use of unequivocal and readily available predictors, a completely automated use of our model is conceivable. Future work will focus on the external validation of our model using data from different European hospitals.

## Conclusions

The presented models can be used to accurately predict the probability of nasal carriage of MRSA and the expected PPV of a commercial molecular test. Model output can be used to inform a variety of clinically relevant decisions including empiric treatment of infections (e.g. choice of antibiotics), infection control (e.g. prompting of preemptive isolation), and choice of screening method (e.g. by restricting rapid tests to indiviudals with expected PPV ≥ 80%). The use of unequivocal predictors potentially permits automated scoring of data extracted from existing hospital information systems.

## Abbreviations

AUC: Area under the curve; BDGO: Becton-Dickinson’s Rapid Diagnostic Test “GeneOhm”; BHI: Brain-heart infusion; CI: Confidence interval; DC: Derivation cohort; ICD: International Classification of Diseases; ICD-10-GM: 10^th^ version of ICD, German Modification; ICU: Intensive care unit; IVC: Internal validation cohort; MRSA: Methicillin-resistant *Staphylococcus aureus*; NPV: Negative predictive value; OR: Odds ratio; PCR: Polymerase chain reaction; PPV: Positive predictive value; RDT: Rapid diagnostic test; ROC: Receiver operating characteristic; SCCmec: Staphylococcal cassette chromosome *mec*; Sn: Sensitivity; Sp: Specificity.

## Competing interests

The authors declare that they have no competing interests.

## Authors’ contributions

JE selected the internal validation cohort, analyzed the data, and drafted the manuscript; PUH contributed to data analysis and interpretation; CS validated the rapid diagnostic test; FE provided data pertaining to the internal validation cohort; HB, SM, AKM, HR, WM, CWe, and CWu supervised and monitored administration of the rapid diagnostic test; MF contributed to the design of the prevalence study; UV designed the prevalence study (derivation cohort), and supervised the validation of the rapid diagnostic test. All authors critically revised and approved the final manuscript.

## Pre-publication history

The pre-publication history for this paper can be accessed here:

http://www.biomedcentral.com/1471-2334/13/111/prepub
